# Protective effects of GABA against metabolic and reproductive disturbances in letrozole induced polycystic ovarian syndrome in rats

**DOI:** 10.1186/s13048-017-0359-7

**Published:** 2017-09-15

**Authors:** Asad Ullah, Sarwat Jahan, Suhail Razak, Madeeha Pirzada, Hizb Ullah, Ali Almajwal, Naveed Rauf, Tayyaba Afsar

**Affiliations:** 1 Department of Animal Sciences, Quaid-i-Azam University, Islamabad, Pakistan; 2 Department of Community Health Sciences, College of Applied Medical Sciences, King Saud University, Riyadh, Saudi Arabia; 3 Department of biochemistry, Quaid-i-Azam University, Islamabad, Pakistan

**Keywords:** Gamma amino butyric acid, Follicles, Cysts, Oxidative stress, ELISA

## Abstract

**Background:**

PCOs is a heterogeneous disorder with anovulation/oligo ovulation usually taken as oligo menorrhoea or amenorrhoea, hyperandrogenemia, hirsutism, acne, androgen alopecia and polycystic ovaries as the key diagnostic feathers. The study was undertaken to investigate the possible protective and ameliorating effects of GABA in Letrozole induced PCOS model in rats by targeting insulin resistance.

**Methods:**

PCOs in Adult female rat was induced by the daily gastric administration of letrozole (1 mg/kg/day) in CMC (0.5%) for 36 days. Rats were given metformin (2 mg/kg), GABA (100 mg/kg/day) and GABA (500 mg/kg/day) along with letrozole. One group severed as vehicle control. On the 37 day, the animals were euthanized, and anthropometrical, biochemical (glucose, insulin, lipids, testosterone, Estradiol, Progesterone, oral glucose tolerance test, total protein content in ovary, cholesterol level, triglyceride, HDL, LDL), Antioxidants (CAT, POD, GSR, ROS, GSH, TBARS), and histopathological evaluation of ovaries were carried out. Daily colpocytological examination was also carried out until the termination.

**Results:**

Both the doses of GABA significantly reduced body weight, body mass index and testosterone. While the levels of CAT, SOD, POD and Estradiol (E_2_) were significantly increased in the both doses of GABA. A favourable lipid profile, normal glucose tolerance, and decreased in the percentage of estrus smears were observed. Histopathological examination of ovary revealed a decreased in the number of cystic follicles, and decreased in the adipocytes respectively. The effects observed with GABA were comparable to that with metformin.

**Conclusion:**

The results suggest that GABA treatment has shown protective effect in PCOs and provide beneficial effect either by reducing insulin resistance or by inducing antioxidant defence mechanisms.

## Background

The polycystic ovary syndrome (PCOs) is one of the most common endocrine disorder present in women worldwide. PCOs is a heterogeneous disorder with anovulation/oligo ovulation usually taken as oligo menorrhoea or amenorrhoea, hyperandrogenemia, hirsutism, acne, androgen alopecia and polycystic ovaries as the key diagnostic feathers [1, 2]. There is also a great deal of chance for the development of metabolic and cardiovascular abnormities because of the presence of insulin resistance (IR) as the central pathogenic feature [3].

Obesity and PCOs seems to be in close relation as many family studies have shown that weight gain promotes the chances of PCOs [4]. Lower levels of sex hormone binding globin (SHBG) are also observed [5]. Insulin resistance (IR) is main defining characteristic of PCOs, occurs in 50–70% of the population. Many of the scientists have also suggested that there is a big link between insulin levels and androgens [5]. Insulin stimulates thecal cells to produce androgens, and the higher levels of androgens are related to many problems as the symptoms seen in PCOs. The hyperinsulinemia which is observed in PCOs is mostly a result of increased secretion of basal insulin along with decreased hepatic insulin clearance [6]. The relationship has been supported by research which stated that reduced androgens were also seen with improvements in insulin sensitivity [7].

Inappropriate gonadotropins secretion is associated with the very classical forms of PCOs. Women with PCOs exhibit a high LH secretion with very low level of FSH secretion [8]. Premature androgen production may also explain the arrested antral follicle development in PCOs [9].

Hyperinsulinemia is a key element in the pathogenesis of PCOS. Insulin sensitizing drugs including metformin and thiazolidinediones (TZDs) have been used as a treatment for this syndrome [10]. It prevents hepatic glucose making and improves peripheral tissue sensitivity to insulin, reducing the androgen synthesis by ovarian theca cells [11]. Metformin also suppresses ovarian steroidogenesis [12, 13].

GABA exerts protective and regenerative effects on islets beta cell and reverses diabetes. Outside of the brain, GAD and GABA receptors have been reported in the pancreatic islets, the gastrointestinal tract, ovaries and adrenal medulla. Treatment with GABA improves glucose tolerance and insulin sensitivity by inhibiting inflammation in fat fed mice [14]. GABA has been reported as a positive regulator of antioxidant enzymes, reduces ROS and reducing cholesterol and triglycerides in the human [15, 16]. The present study was design to investigate the possible protective and ameliorating effects of GABA in Letrozole induced PCOS model in rats by targeting insulin resistance that can effects Glucose levels in PCOS, antioxidants status of the ovaries, production Reactive oxygen species (ROS) in the ovaries, synthesis and secretion of steroid hormones, follicle development and folliculogensis and cholesterol and plasma creatinine levels.

## Methods

The study was conducted in the department of animal Sciences, Quaid-i-Azam University, Islamabad, Pakistan and the study was approved by the ethical committee of department of animal sciences and the work was performed in the months of March–May. Adult female rats, weighing 200 ± 15 g were obtained from the department animal facility of Quaid-i-Azam University, and were kept under 12/14 h light/dark cycle in 22–25 °C temperature. Animals were provided with standard laboratory food and tap water. Vaginal smear was examined daily for estrous cyclicity and the animals having two regular cycles were selected and included in the study. Letrozole (Femara) was purchased from novartis. Metformin (Glucophage) was purchased from merck Serrano. Gamma-Aminobutyric acid (GABA) was purchased from International Labortory, USA and stored at room temperature. Enzyme linked immunosorbent assay (ELISA) kits for estradiol (E_2_), testosterone (T) and progesterone (P_4_) were purchased from Microlisa (Amgenix USA). Animals (*n* = 25) were divided into five groups according to the treatment. Group 1 animals were control group and were treated with 0.5% corboxymethylcellulose (CMC) through gastric intubation daily throughout the experiment. Animals in group 2 were allocated as PCOs group and received daily gavage of 1 mg/kg/day Letrozole dissolved in 0.5% CMC throughout the experiment. Animals in group 3 received letrozole (1 mg/kg.day) and co-treatement with metformin (2 mg/kg) from day 21 of treatment till the end of experiment. This group was allocated as metformin group. Group 4 animals were treated with daily dose of Letrozole (1 mg/kg/day) and GABA (100 mg/kg/day) from day 21 till the end of experiment. Animals in group 5 received daily dose of Letrozole (1 mg/kg/day) and were treated with GABA (500 mg/kg/day) from day 21 till the end of the experiment.

All the animals were anesthetized with chloroform on day 37, 24 h after the treatment. Blood samples were obtained by cardiac puncture in heparinized syringes and centrifuged at 3000 rpm for 10 min. Plasma was separated and placed at −20 °C until analyzed. Ovaries were removed, weighed and ovarian volume was measured. Left ovaries was placed at −80 °C for analysis of antioxidant enzymes while right ovaries were washed in cold physiological saline and placed in 10% formalin for histological processing. In tissue histology ovaries were placed in 10% formalin for 48 h for fixation. After fixation, tissues were subjected to ascending grades of alcohol for dehydration followed by two washes in xylene for clearing. Tissues were checked for clearance and then embedded in paraffin wax after words the fixed tissues were embedded for microtomy. In the process of microtomy and section cutting 7 micrometer thick sections were obtained from tissue. Every tenth section of the tissue was cut out of the ribbon and placed in warm water at low levels for stretching. Sections were carefully placed on albumenized slides, air dried for 30 min and transferred into paraffin oven for 1 h at 37–40 °C. For tissue staining sections were de-parafinized by placing the slides in xylene for 10 min and xylene washes were changed twice (10 min each). After complete deparaffinization, slices were subjected to descending grades of alcohol for rehydration. Destaining was done when needed with 95% Ethanol and cover slips were placed on the slides. For antioxidant enzymes the ovarian tissues were homogenized in 2 ml of phosphate buffer saline (PBS), centrifuged at 4 °C for 30 min at 3000 rpm. Supernatant was separated and was used for estimation of antioxidant enzymes, protein content and lipid profile.

In antioxidant enzymes Catalase (CAT) activity was determined by the decrease in absorbance due to H_2_O_2_ consumption by method of Aebi. [17]. Peroxidase (POD) activity in homogenate was determined by spectrophotometric method of [18]. Glutathione reductase (GSR) activity was determined by the method of [19]. Reactive oxygen species (ROS) activity was determined by following method of [20]. Reduced glutathione (GSH) activity was determined by following method of [21] with some modifications by utilizing Ellman’s reagent (DTNB).

Estimation of Lipid per oxidation by TBARS was determined by the malondialdehyde in homogenate by reaction with thiobarbituric acid (TBA) by the method of [22].

Total protein content in ovarian tissue was quantitatively determined by using specific protein kit. The kit was purchased from AMEDA Labordiagonstik GmbH Krenngasse, Graz/Austria. Plasma cholesterol level was determined by using AMP diagnostic kit (AMEDA labordiagnostik Gmbh, Austria) and was analyzed on picco 5 chemistry analyzer. Plasma triglyceride levels were determined by using AMP diagnostic kit (AMEDA labordiagnostik Gmbh) and were analyzed on picco 5 chemistry analyser. Plasma HDL Cholesterol level was determined by using AMP diagnostic kit (AMEDA labordiagnostik Gmbh) and were analyzed on picco 5 chemistry analyser. Plasma LDL Cholesterol level was determined by using AMP diagnostic kit (AMEDA labordiagnostik Gmbh) and were analyzed on picco 5 chemistry analyser.

For the hormonal analysis three different kits were purchased form (Amgenix Inc., USA) for the determination of testosterone, Estradiol and progesterone from serum. Testosterone concentrations were quantitatively determined by using EIA tests kits (Amgenix Inc., USA). Estradiol EIA tests kits (Amgenix Inc., USA) were used to determine E_2_ concentrations (pg/ml) in the serum. Progesterone (P4) EIA tests kits (Amgenix Inc., USA) were used to determine P4 concentrations (ng/ml) in the serum.

All the data were expressed as Mean ± SEM. One way Analysis of Variance (ANOVA) followed by tukey’s test was used for comparing different groups using graph pad prism 5 software. Probability value less than 0.05 was considered statistically significant.

## Results

Protective effect of different doses of GABA against Letrozole induced Polycystic Ovarian syndrome was determined by using different parameter.

### Determination of estrous cyclicity

**Fig. 1:**
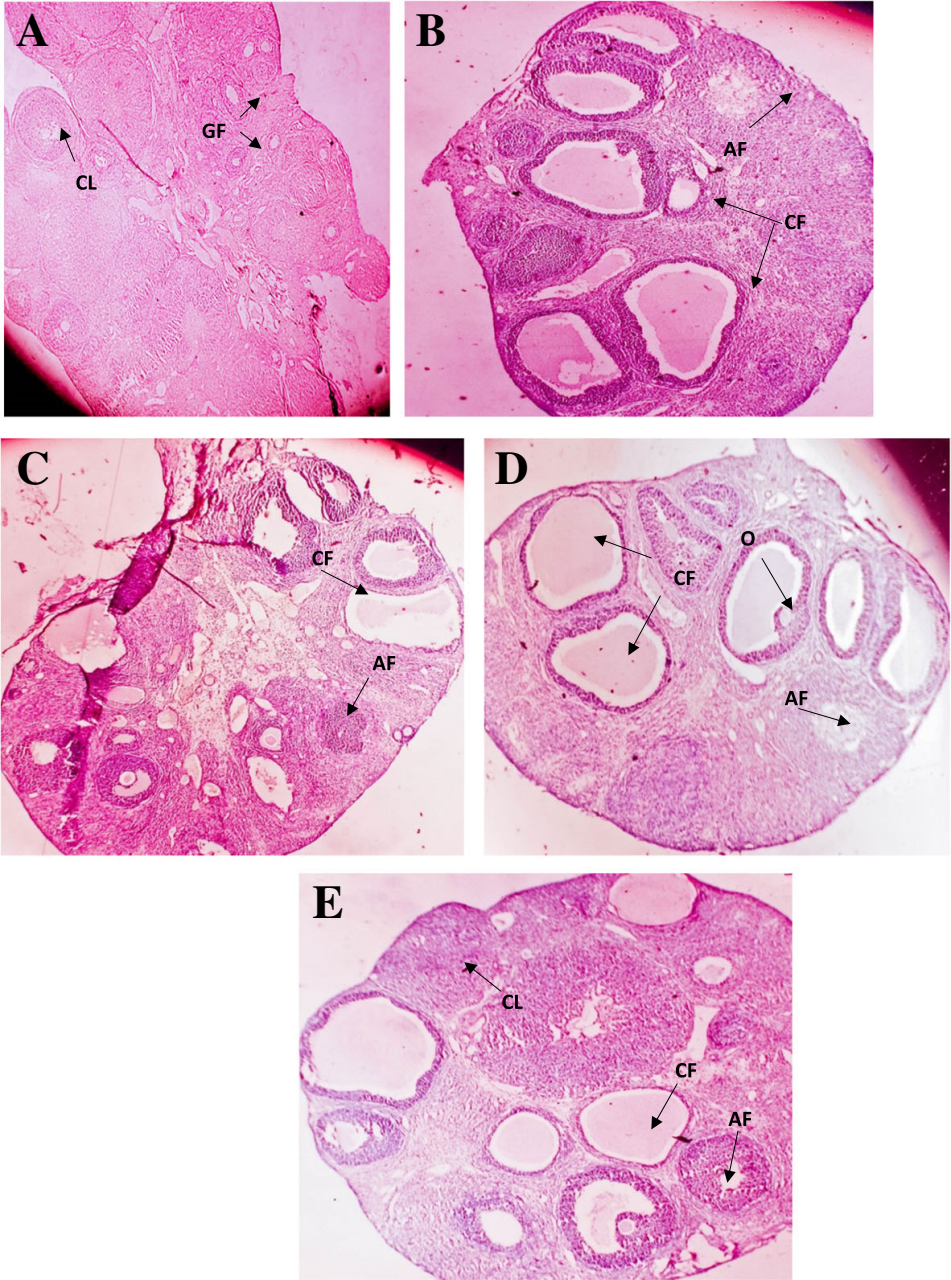
Histopathological features of ovary in Letrozole induced PCOS in rats and (**a**) Control group (**b**) PCOS group (**c**) Metformin group (**d**) GABA 1 (**e**) GABA 2. Corpus Luteum (CL), Growing follicles (GF), Oocyte (O), Atertic follicle (AF), Cystic follicle (CF) (4X magnification)

Estrous cycle was studied by regular monitoring and collection of vaginal smear from all the experimental groups throughout the experiment. Estrous cycle in the PCOS group was irregular and long as compared to the control animals while the metformin and GABA treated groups showed normal estrous cycle. Changes in the Estrous cycle are represented in Fig. 1.

### Body weight and blood glucose

Animals included in the study were of approximately equal initial body weights (Table 1). However, final body weights showed significant difference in PCOS when comparison was made with control and other treated groups. Significant increase in body weight was observed in PCOS group (*P* < 0.001) as compared to control group. Significant difference was also being observed in metformin treated group (*p* < 0.05), GABA 1 treated group (*P* < 0.01) and GABA 2 treated group (P < 0.01) as compared to the PCOS group (Table 1).

**Table 1:** Mean ± SEM of body weight, glucose level, body mass index, body mass gain, ovarian diameter, abdominal and thoracic circumference in control, PCOS, metformin, GAB 1 (100 mg/kg) and GABA 2 (500 mg/kg) treated groups before and after 36 days of experiment

Parameters	Control	PCOS	Metformin	GABA1	GABA2
Initial Body Weight (gm)	156 ± 01.37	156 ± 05.38	157 ± 07.57	150 ± 05.79	157 ± 04.22
Final Body Weight (gm)	181 ± 04.11	260 ± 02.81***	203 ± 03.29+	210 ± 04.57**++	222.2 ± 02.15**++
Initial Glucose (mg/dL)	117.20 ± 01.93	103.20 ± 02.81	122.41 ± 03.29	113.00 ± 04.57	120.40 ± 02.15
Final Glucose (mg/dL)	120.22 ± 03.21	166.46 ± 20.00***	135.21 ± 01.91++	129.00 ± 05.08+	138.40 ± 06.20+
Body mass Index (g/cm2)	0.49 ± 0.01	0.54 ± 0.02	0.47 ± 0.01	0.72 ± 0.03	0.69 ± 0.04
Body mass gain (g/kg)	4.34 ± 0.94	27.76 ± 8.61	16.21 ± 5.04	29.66 ± 6.47	15.17 ± 7.21
Right Ovary diameter (mm)	4.80 ± 00.37	5.10 ± 01.04	4.60 ± 00.25	6.10 ± 00.96	5.80 ± 00.12
Left Ovary diameter (mm)	5.00 ± 0.44	5.60 ± 0.24	5.20 ± 0.20	6.00 ± 0.15	5.70 ± 0.20
Abdominal Circumference (cm)	1.06 ± 0.09	1.05 ± 0.31	1.06 ± 0.51	1.04 ± 0.44	1.09 ± 0.34
Thoracic circumference (cm)	1.06 ± 0.23	1.05 ± 0.10	1.06 ± 0.18	1.16 ± 0.29	1.22 ± 0.24

Values are expressed as Mean ± SEM

*, **, *** indicate significance from the control group at *P* < 0.001, 0. 01 and 0.05 probability level

+, ++, +++ indicate significance from the PCOS group at P < 0.001, 0. 01 and 0.05 probability level

Blood glucose level as determined at day 1 of the experiment were same in all the groups, but Final blood glucose levels showed significant difference in PCOS when comparison was done with control and other treated groups (Table 1). There was Significant increase (P < 0.001) observed in blood glucose of PCOS group as compared to control group. In PCOS group blood glucose was significantly different as compared to metformin treated group (*p* < 0.01), GABA 1 and GABA 2 treated group (*P* < 0.05) and presented in Table 1.

### Body length and body mass index (BMI)

There was significant increase (*p* < 0.05) in body length of PCOS group when compared with control group but there was no significant difference observed in the body length of metformin, GABA 1 and GABA 2 treated groups as compared to PCOS group. BMI was not significantly different in all treatment groups and presented in Table 1.

### Ovarian diameter, abdominal circumference (AC) and thoracic circumference (TC)

There were no significant differences noticed in ovarian length, abdominal circumference and thoracic circumference in all the treated groups as compared to control group (Table 1).

### Total protein, CAT, SOD and POD

Total protein content showed no significant difference in all treated groups. CAT and SOD content of the ovarian tissues in PCOS groups were significantly low (*P* < 0.01) as compared to the control group. It was also observed that the Metformin treated group showed a good recovery of CAT (*P* < 0.05) and SOD (*P* < 0.001) as compared to PCOS group. CAT and SOD levels were significantly high (P < 0.05, 0.001) in GABA 2 treated groups as compared to PCOS, but there was no significant difference observed in GABA 1 treated group (Table 2). POD activity in PCOS group was significantly reduced (P < 0.05) as compared to the control group. GABA 2 group has significantly high levels of POD (*P* < 0.001) but GABA 1 did not increased POD activity as compared to PCOS group (Table 2).

**Table 2:** Mean ± SEM of total protein, CAT, SOD, POD, GST, GSR, GSH, T-BARS, ROS, cholesterol, triglycerides, HDL, testosterone, progesterone and estradiol concentrations in control, PCOS, metformin, GAB 1 (100 mg/kg) and GABA 2 (500 mg/kg) treated female rats after 34 days of experiment

Parameters	Control	PCOS	Metformin	GABA1	GABA2
Total Protein (mg/g)	2.55 ± 0.23	3.09 ± 0.41	2.77 ± 0.26	2.04 ± 0.05	2.65 ± 0.26
CAT (u/mg)	29.44 ± 2.60	23.41 ± 4.06**	22.63 ± 2.35+	17.05 ± 2.28*	26.94 ± 2.28+
SOD (u/mg)	1.21 ± 0.24	0.99 ± 0.13**	1.90 ± 0.21+++	1.48 ± 0.11+	2.63 ± 0.37*+++
POD (nmole)	0.09 ± 0.16	0.04 ± 0.07*	0.47 ± 0.02+++	0.12 ± 0.09	0.18 ± 0.38++
GST (u/mol/mg)	61.14 ± 11.39	65.82 ± 03.13	55.48 ± 08.27	72.95 ± 17.41	59.31 ± 23.72
GSR (u/mol/mg)	174.36 ± 8.90	29.23 ± 13.30**	162.38 ± 16.60+++	97.66 ± 12.94++	161.09 ± 12.89+++
GSR-PX (u/mol/mg)	24.86 ± 02.97	10.71 ± 04.41	54.30 ± 05.55**+++	48.03 ± 08.81*+++	22.57 ± 03.04
GSH (u/mol/mg)	405.74 ± 36.39	558.79 ± 95.65	405.23 ± 27.63	359.62 ± 06.16	495.53 ± 50.12
T-BARS (nmol/mg)	206.46 ± 33.81	436.70 ± 63.74**	268.63 ± 56.98++	293.39 ± 24.38	245.63 ± 73.20+
ROS (u/min)	39.47 ± 13.63	79.19 ± 11.20*	38.14 ± 03.53+	18.78 ± 11.51+++	12.18 ± 08.60+++
Cholesterol (mg/dL)	215.60 ± 3.43	231.10 ± 4.27*	211.30 ± 6.81	222.21 ± 2.53+	210.10 ± 3.34++
Triglycerides (mg/dL)	185.11 ± 4.23	213.10 ± 5.16**	197.40 ± 5.82	204.20 ± 5.93	190.12 ± 6.32+
HDL (mg/dL)	160.70 ± 4.17	144.41 ± 3.63	153.21 ± 1.80	156.43 ± 5.52	165.34 ± 4.24+
Testosterone (ng/ml)	1.24 ± 0.14	2.07 ± 0.14**	1.24 ± 0.17++	1.50 ± 0.20	1.35 ± 0.10++
Progesterone (ng/ml)	6.96 ± 0.10	6.70 ± 0.15	6.82 ± 0.07	6.63 ± 0.11	6.70 ± 0.21
Estradiol (pg/ml)	4.89 ± 0.14	2.79 ± 0.18*	4.40 ± 0.17+	2.36 ± 0.24	4.46 ± 0.46++

Values are expressed as Mean ± SEM

*, **, *** indicate significance from the control group at P < 0.001, 0. 01 and 0.05 probability level

+, ++, +++ indicate significance from the PCOS group at *P* < 0.001, 0. 01 and 0.05 probability level

### GST, GSR and GSR-PX

There was no significant difference in the GST activity in all the treated groups as compared to the control group. GSR activity was significantly reduced (P < 0.001) in PCOS group as compared to control group but in metformin treated group, GSR activity was significantly high (P < 0.001) as compared to PCOS group. Similarly in GABA 1 and GABA 2 treated groups, GSR activity was significantly high (*P* < 0.01 and P < 0.001 respectively) when compared to the PCOS group (Table 2). There was significant difference observed in GSR-PX activity of PCOS and control group. In GABA 1 and Metformin treated groups, GSR-PX values were significantly high (P < 0.001) as compared to PCOS group but GABA 2 did not show any effect on GSR-PX activity as compared to the PCOS group (Table 2).

### GSH, T-BARS and ROS

There was no significant change observed in the GSH levels in PCOS and in all the other groups (Table 2). The Mean ± SEM of T-BARS were significantly high (P < 0.01) in the ovarian tissues of PCOS group as compared to the control group, while Metformin treatment significantly reduced (*P* < 0.01) in ovarian T-BARS levels as compared to the PCOS group. The ovarian T-BARS levels in the GABA 2 treated group were significantly reduced as compared to the PCOS group (Table 2). Reactive oxygen species (ROS) in the ovarian tissues were determined and was found significantly high (*P* < 0.05) in PCOS group as compared to the control group. Metformin and GABA treatment (1 and 2) resulted in significant reduction in the levels of ROS (P < 0.05 and *P* < 0.001) when comparison was made with PCOS group (Table 2).

### Cholesterol, triglyceride and HDL

Cholesterol concentrations (mg/dL) in PCOS group was significantly high (P < 0.05) as compared to control group, while they were low in Metformin treated group. GABA 1 and GABA 2 treatment caused a significant decrease in cholesterol concertation as shown in Table 2.

The levels of Triglycerides in PCOS groups were significantly high (*P* < 0.01) as compared to the control group. While Metformin and GABA 1 treatment caused no reduction in triglycerides concentration however GABA 2 did cause a significant difference as compared to PCOS (Table 2).

HDL concentrations did not change in PCOS, metformin and GABA 1 treated groups; however, GABA 2 treatment caused significant increase in HDL concentration as compared to PCOS group (Table 2).

### Hormonal analysis

Plasma testosterone concentration was measured because letrozole has been reported to cause hyperandrogenism that leads to PCOS. Testosterone concentration in the PCOS group was significantly high (P < 0.01) as compared to the control group. Testosterone concentrations in metformin and GABA 2 was significantly low (P < 0.01) as compared to the PCOS group. Plasma progesterone concentration was not affected in all the treated groups. However, Estradiol concentration was significantly reduced (*P* < 0.05) in PCOS animals as compared to the control group. However, GABA 2 group exhibited significantly high (P < 0.01) levels of Estradiol as compared to the PCOS group (Table 2).

### Histological results

Number of different types of follicles were counted and presented in Table 3. Two methods were used for counting of different types of follicle. They were counted on the basis of diameter and type of follicle i.e. cystic, fibrotic and corpus leuteum [23].

**Table 3:** Mean ± SEM number of ovarian follicles in control, PCOS, metformin, GAB 1 (100 mg/kg) and GABA 2 (500 mg/kg) after 34 days of experiment

Developing follicles (μm)	CONTROL	PCOS	METFORMIN	GABA 1	GABA 2
D 20–60	8.66 ± 0.55	9.00 ± 0.73	7.16 ± 0.70	9.66 ± 0.55	8.50 ± 0.50
D 60–100	7.60 ± 0.50	7.60 ± 0.92	7.20 ± 0.66	5.20 ± 0.91	5.20 ± 0.58
D 100–200	7.00 ± 0.70	3.00 ± 0.54*	8.20 ± 1.06	3.80 ± 0.80	4.80 ± 0.37
D 200–400	8.00 ± 0.70	4.60 ± 0.74	6.60 ± 1.07	4.80 ± 0.73	4.20 ± 0.73
D 400–600	2.20 ± 0.37	0.80 ± 0.58	1.00 ± 0.44	0.80 ± 0.37	0.60 ± 0.24
>600	3.20 ± 0.37	0.80 ± 0.58	0.60 ± 0.24	0.40 ± 0.24	1.20 ± 0.37
Cystic Follicle	0.00 ± 0.00	9.17 ± 0.70 **	5.16 ± 0.30++	6.16 ± 0.47++	7.33 ± 0.42 +
Corpus Luteum	8.80 ± 0.37	2.40 ± 0.24 ***	4.20 ± 0.58 +	2.40 ± 0.50 ++	4.40 ± 0.24 ^++^
Atretic Follicle	4.00 ± 0.25	3.50 ± 0.34 ^**+++^	2.83 ± 0.47	2.66 ± 0.33	2.83 ± 0.40 +

Values are expressed as Mean ± SEM

*, **, *** indicate significance from the control group at P < 0.001, 0.01 and 0.05 probability level

+, ++, +++ indicate significance from the PCOS group at *P* < 0.05, *P* < 0.01 and P < 0.001 probability level

### Follicles with diameter 20–60, 60–100 μm

Mean ± SEM number of follicles with 20–60 and 60–100 μm diameters were not significantly different in different groups (Table 3).

### Follicles with diameter 100–200, 600 and > 600 μm

The mean number of ovarian follicle with 100–200 μm diameter were significantly reduced (P < 0.05) in PCOS as compared to the control group. There was no significant increase noticed in GABA treated groups. Follicles with diameter ranging from 200 to 600 μm in all treated groups were found with no significant difference. The mean numbers of ovarian follicle was greater than 600 μm were non-significantly different in PCOS, Metformin and GABA treated groups (Table 3).

### Cystic follicle

There was a significant decrease (*p* < 0.001) found in the number of cystic follicles of Metformin group as compared to PCOS. It was also observed that the number of cystic follicles in GABA 1 and 2 treated groups were significantly decreased (p < 0.001) as compared to PCOS group (Table 3).

### Atretic follicle

PCOS group showed a highly significant (p < 0.001) increase in number of atretic follicles as compared to control group. There was Significant (*p* < 0.05) decrease observed in the number of atretic follicles in Metformin GABA 1 and GABA 2 treated groups as compared with PCOS group (Fig. 1).

### Corpus luteum

The Number of Corpus Leuteum significantly reduced (*p* < 0.001) in PCOS group as compared to control group. There was also no significant (p < 0.001) difference found in in the number of Corpus Leuteum of Metformin and GABA 2 treated groups as compared to PCOS group (Table 3).

## Discussion

The present study was designed to investigate the possible beneficial effects of GABA in mitigating PCOS by targeting insulin resistance in letrozole induced PCOS model of albino rats. There have been studies which show that outside of the brain, GAD and GABA receptors have been reported in the pancreatic islets, the gastrointestinal tract, ovaries and adrenal medulla. On the basis of previous studies, we targeted insulin resistance via oral administration of GABA to treat letrozole induced PCOS in rats. Results of present study showed normal glucose levels in GABA and metformin treated groups while glucose levels were elevated in the PCOS group. These findings have provided base for the study of further complications related to PCOS and insulin resistance.

Antioxidant enzymes provide the first line defence mechanism which prevents biological molecules (lipids, proteins, DNA) from damage by inhibiting ROS formation. Hydrogen peroxide (H_2_O_2_), nitric oxide (NO), superoxide anion (O_2_^−^) and hydroxyl radical (OH) are central reactive oxygen and nitrogen species that are involved in tumorigenesis and mutagenesis. Superoxide dismutase (SOD) counteracts toxic effects of superoxide anion. Levels of antioxidant enzymes are important because dismutation of superoxide anion to form H_2_O_2_ is catalysed by SOD while H_2_O_2_ is converted to water molecules by the activity of catalase (CAT) and glutathione peroxidase (GSH-PX) while reduced glutathione (GSH) is used as an electron donor in such reactions [24]. Similarly, GSH levels are retained by thiol containing non-protein compound called glutathione reductase (GSR). GSR regenerates GSH (reduced form) from GSSG (oxidized form) for the constant activity of GSH-PX [25]. In PCOS group, SOD, POD, and CAT were significantly reduced as compared to the control group; similarly, POD activity was also low in PCOS as compared to the control group. However GABA and metformin treatment significantly recovered activity of CAT, SOD and POD in letrozole induced PCOS model. GSH levels were not different in PCOS group and were similar to control, however, GSR and GSR-PX levels were reduced in PCOS. Metformin treatment and GABA treatment significantly induced levels of GSR and GSR-PX levels suggesting the protective effect of GABA as described previously [15, 16].

NADPH oxidases are specialized enzymes that can generate superoxide anion which can be eliminated by CAT, POD and SOD, decreasing LPO in order to protect spermatozoa from oxidative stress [26]. In the PCOS, reduced levels of antioxidants have been reported previously and have been linked to the female infertility [27]. Similarly GABA has been reported as an inducer of macrophages infiltration and induces antioxidant enzymes activity [15, 16]. Similarly antioxidant potential and antioxidant enzymes inducer effect of GABA has been reported previously, by reducing ROS in the tissues and neurons. In present study, antioxidant enzymes levels were reduced in the PCOS group as compared to the control animals. Similarly, ROS was high in PCOS but less in control groups. Interesting results were observed when comparison was made between metformin treated groups, GABA treated groups and PCOS animals. Metformin and GABA treatment not only involved in reducing ROS level in the ovarian tissues but also induces antioxidant enzymes and glutathione levels in the ovarian tissues. This effect of GABA may be due to the effect of GABA on antioxidant recovery through overcoming insulin resistance or effecting primary fatty acid amide (pFAA) disorder which is caused by insulin resistance. High levels of lipid peroxidation as observed in the PCOS group were also recovered by treatment with metformin and GABA. These results are in consistent with previous studies in which GABA treatment reduced ROS and induced antioxidant enzymes in sperm and Roman hens [1, 2]. Similarly, insulin resistance leads into disruption of antioxidant status of the body besides hyperglycemia and obesity, which are also major causing agents that disrupts body defense mechanism [28, 29]. It was reported that GABA treatment for 20 weeks restored glucose levels and muscular oxidation by treating hyperglycemia and pFAA disorder in mice. Cholesterol level was significantly high in the PCOS group as compared to the control; however GABA treatment significantly reduced Cholesterol level. These results are in accordance with the previous study [30], reported that treatment with GABA reduces cholesterol levels in chronic ethanol treated rats. However triglycerides were not significantly changed in all treated groups. Interestingly, HDL levels were significantly high by (500 mg/kg) dose of GABA. Elevated levels of triglyceride were found in men who consumed GABA enriched white rice while HDL levels were not affected. Similarly cholesterol levels were found low in men who consumed white rice [31].

High levels of testosterone in PCOS, as an indicator of the disorder, were significantly reduced by metformin and GABA 2 treatment, while low level of estradiol in PCOS group were induced by treatment with metformin and GABA however, progesterone levels were not affected. These results suggest the healing of the ovarian tissues and reduction in the number of cysts in the tissues. Similarly, increase in number of healthy follicles may be induced by GABA treatment. These results can be supported by the normal levels of glucose found in metformin and GABA treated groups because insulin resistance is the major contributory factor in altering hormonal levels among PCOS patients.

Finally, histological observations supported the protective and beneficial effect of GABA in letrozole induced PCOS model of rats. Number of cystic follicles was reduced in GABA treated groups as compared to PCOS group same as in metformin treated group. Number of atratic follicles was also reduced in metformin and GABA treated groups as compared to the PCOS group. Number of corpus luteum was increased in metformin and GABA treated groups as compared to the PCOS group.

## Conclusion

In conclusion present findings demonstrate the antiandrogenic properties of GABA in treating PCOS. GABA also protected the ovarian tissue and prevented cyst development in this study. This effect of GABA may be because of inducing first line defence mechanism of the body and by reducing insulin resistance that improves the reproductive health by acting on the ovarian tissues. However, GABA also showed estrogenic and antiandrogenic effects by recovering the ovarian cysts which could be supportive in managing PCOS. GABA also showed to boost the antioxidant status by reducing the oxidative stress, lipid peroxidation and ROS..It displayed a significant role in subsiding the hyperlipidemic state and hyperglycemic state and it can be used as a possible ameliorative medication for curing clinical and biochemical characteristics of polycystic ovary syndrome. Further studies are required to investigate the exact mechanism of action of GABA in PCOS Rats model and also to investigate the possible role of GABA in curing PCOS management in women so that it can be used as an adjunct therapy for the treatment pf PCOS.

## Abbreviations

17 – β –E_2_: 17 - β -estradiol
AC: Abdominal circumference
BMI: Body mass index
DPX: Di –n-butyl phthalate in xylene
E_2_: Estradiol
ELISA: Enzyme linked immunosorbent assay
ER: Estrogen receptor
FSH: Follicle Stimulating Hormone
GABA: Gamma-Aminobutyric acid
GnRH: Gonadotrophin releasing hormone
HRP: Horseradish peroxidase
LH: Luteinizing Hormone
Ml: Milli liter
PCOS: Poly Cystic Ovarian Syndrome
ROS: Reactive Oxygen Specie
T: Testosterone
TC: Thoracic circumference
TMB: Tetra methyl benzidine
Μl: Micro liter
Μm: Micro meter
